# A Study on the Spatial Pattern of the Ecological Product Value of China’s County-Level Regions Based on GEP Evaluation

**DOI:** 10.3390/ijerph20043181

**Published:** 2023-02-11

**Authors:** Ping Shen, Lijuan Wu, Ziwen Huo, Jiaying Zhang

**Affiliations:** 1 Guangzhou Urban Planning & Design Survey Research Institute, Guangzhou 510060, China; 2 Guangdong Enterprise Key Laboratory for Urban Sensing, Monitoring and Early Warning, Guangzhou 510060, China

**Keywords:** GEP evaluation, spatial pattern, ecological product value, China’s county-level regions

## Abstract

Gross Ecosystem Product (GEP) is a concept that reflects ecological product value by using geospatial technology. It can demonstrate the spatial distribution of ecological products and provide new perspectives and refined support for spatial planning. China’s county-level regions are important units for the promotion of ecological product value. Based on the concept of GEP, this study evaluated the ecological product value of China’s county-level regions in 2020, used Local Indicators of Spatial Association (LISA) to visualise spatial patterns and conducted a correlation analysis between the GEP indices and economic and land use factors. The study found that the results of evaluation and analysis varied by spatial distribution: (1) county-level regions with high provisioning service indices are concentrated in northeastern China and southeastern China; (2) county-level regions with high regulating service indices are concentrated south of the Yangtze River and in the southern region of the Qinghai-Tibet Plateau; (3) county-level regions with high cultural service indices are concentrated in southeastern China; (4) county-level regions with high composite GEP indices are concentrated in northeastern China. The results have different correlations with different factors, reflecting the complex mechanisms behind ecological value transformation. For example, the composite GEP index for an area has a strong positive correlation with the area’s proportions of woodland area, water area and GDP.

## 1. Introduction

Ecological product value includes the value of various final material goods and services that ecosystems provide for human well-being as well as for economic and social development. Since the late 1960s, the value of ecosystem services has been highly emphasized [1] as a tool for coordinating ecological protection and economic development, correcting the neglect of ecosystem services in traditional decision-making processes [2] and providing important support to governments when making integrated economic, social and ecological decisions [3]. The scientific assessment of the value of an ecosystem’s goods and services has become an issue of global concern [4].

The international understanding of ecosystem value has evolved from the “primary production value of resources” to the “value of ecosystem services”. The System of National Accounts (SNA) published by the United Nations in 1953, which takes GDP as its core, also reflected the primary production value of ecological resources but did not show the cost of environmental consumption [5,6]. As the problems of resource shortages and ecological environment deterioration began to gain traction, scholars in the field started to pay attention to economic accounting under the influence of the ecological environment.

In the 1980s, Chinese scholars also began the theoretical and practical exploration of ecosystem services’ value, and Shijun Ma and Rusong Wang first proposed the society–economy–nature complex ecosystem theory. In 1993, the United Nations published the System of Environmental and Economic Accounting (SEEA), which described the stock of environmental assets and its changes with a focus on the economic impact of the consumption of environmental resources. In 1997, Daily and other scholars defined ecosystem services as the natural environmental conditions and utilities formed by ecosystems and ecological processes that sustain human survival [7]. Costanza and other scholars pioneered the quantification of the global ecosystem services based on 17 categories [8], which attracted widespread attention. In 1999, Zhiyun Ouyang and other Chinese scholars started from the functions of ecosystem services to estimate the economic value of the terrestrial ecosystem in China for the first time [9]. They then gradually formed standard specifications for evaluating ecosystem services of single categories at the national level, such as specifications for forest and desert ecosystems. Later, some scholars paid attention to the ecosystem services value (ESV) [10,11]. They studied calculating the value of ecosystems’ services by calculating the areas of various types of land within a specific research scope and determining the ESV-equivalent factors. In addition, relevant research on ecosystem value accounting around the world continues to emerge [12,13,14,15,16,17,18].

Throughout the 21st century, there has been an ongoing series of international projects accounting for the value of ecosystem goods and services, including the UN’s Millennium Ecosystem Assessment (MA); the EU’s The Economics of Ecosystems and Biodiversity (TEEB) project; the World Bank’s Wealth Accounting and the Valuation of Ecosystem Services (WAVES) project; the UN’s System of Environmental-Economic Accounting (SEEA) and Experimental Ecosystem Accounting (EEA) [19]. Among them, the SEEA and EEA are grounded in the perspective of ecosystem assets, which breaks free from the limitations of previous studies that only focused on environmental economic accounting, thereby providing the guidelines and basis for GEP evaluation. In order to establish a methodological system that independently accounts for the value of an ecosystem’s goods and services that can be aligned with national economic statistics, the concept of GEP was first proposed in China in 2012. The calculation idea of the GEP is to calculate the functional quantity of each ecological element and then convert it into value quantity [20]. After receiving initial attention, GEP’s concept connotations, accounting framework and accounting methods were gradually clarified, and a number of local research practices emerged, forming a multi-level, multi-element GEP accounting case at the national [21], provincial [22,23,24,25], city [26,27,28] and county levels [29,30,31,32]. In 2021, the relevant methods of GEP evaluation were incorporated into the System of Environmental and Economic Accounting-Ecosystem Accounting (SEEA-EA) framework by the United Nations.

In general, existing research and practices often focus on the accounting of GEP in a single region, and some studies take provinces and cities as units to carry out horizontal comparisons of GEP. However, the research units in these studies have not been further subdivided, and it is difficult to accurately reflect the differences in the ecosystem service capabilities of different regions. Meanwhile, few studies have paid attention to the extensive spatial pattern of ecological product value and its associated factors, especially economic and land use factors, which makes it difficult to effectively support the government’s comprehensive decision-making.

County-level regions play an essential top-down role in China’s administrative management system and are the core units of China’s ecological civilisation and ecological governance [33]. The varying development conditions and large development gaps across county-level regions complicate high-quality development in China. The GEP evaluation of China’s county-level regions is beneficial for promoting the balanced development of urban and rural areas as well as the coordinated development of the economy and the environment.

Therefore, this study systematically conducted a comprehensive GEP evaluation of the 2020 ecological product value of China’s county-level regions through comprehensive collection of multi-source data and the application of geospatial technologies, and it analysed the spatial distribution characteristics of China’s ecological product value. On this basis, the correlated factors of GEP were analysed from the perspective of land use and economic levels. This analysis supports spatial planning decisions and land resource allocation in multiple dimensions, provides a supporting basis for clarifying the transformation and practical application of ecological value and supports the formulation of ecological governance policies and spatial planning.

## 2. Materials and Methods

### 2.1. Scope of the Study

**Figure 1:**
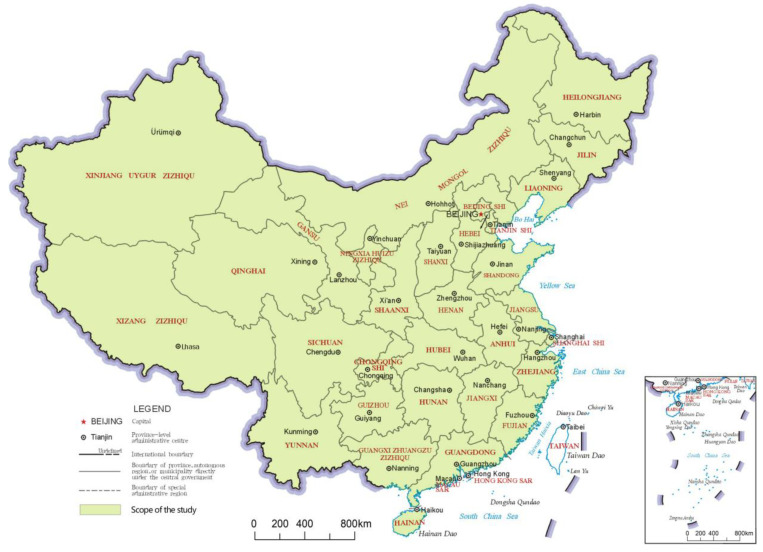
Scope of the Study.

This study covered 2844 county-level regions in China in 2020. The 2844 county-level regions comprised 973 municipal districts, 388 county-level cities, 1312 counties, 117 autonomous counties, 52 banners and autonomous banners, and 2 county-level forest areas and special zones. The scope of the study did not include Hong Kong, Macao and Taiwan in China (Figure 1) because these areas adopt different data statistics methods and different management models of county-level regions than other areas in China. The county-level region is the third region level, according to China’s administrative division, whereas the first is the provincial-level region and the second is the prefecture-level region. Therefore, this study focused on the county level as a research unit, which ensured the research results would have a fine degree of detail.

### 2.2. Research Methodology

#### 2.2.1. Overview of Research Methods

Existing GEP-related research practices are mainly derived from multiple categories of ecosystem service functions for accounting, although the division of specific service functions varies slightly, which is shown in Table 1. Among them, MA, TEEB and other assessment projects propose that the value of ecological product includes four categories of services: provisioning, regulating, cultural and supporting. In 2014, the United Nations published the Experimental Ecosystem Accounting (EEA) manual, which states that supporting services (i.e., intermediate services) are not counted in the calculation, and only the provisioning, regulating and cultural services of ecosystem products with final outputs are included in the scope of ecosystem accounting. This became the basis for the subsequent GEP accounting framework.

In 2020, the Ministry of Ecology and Environment of China issued the “Technical Guide on Accounting for Terrestrial Gross Ecosystem Products”, which proposed that GEP should mainly account for ecological product value directly related to human well-being, including three aspects: provisioning services, regulating services and cultural services. Among them, provisioning services are mainly agriculture, forestry, animal husbandry and fishery products and water resources; regulating services include water conservation, soil conservation, windbreak and sand-fixation, etc.; cultural services include specific indices such as recreation and tourism. There is a basic consensus on the accounting method, and the indices of GEP are calculated through two major steps: biophysical valuation (physical quantity) and ecosystem service monetary valuation. Here, statistics of the bio-physical valuation (physical quantity) are calculated using a physical classification, whereas value valuation mainly adopts the direct market, alternative cost and simulated market methods.

On the whole, although there is an international consensus on the direction of GEP accounting, there are still some differences in the selection of specific indices (Table 1).

#### 2.2.2. GEP Evaluation

GEP reflects the value of various final material goods and services provided by ecosystems for human well-being and economic and social development, and it mainly consists of the value of the provisioning, regulating and cultural services provided by ecosystems. Based on existing standards and norms, the rationality behind the indices and the availability of data, an evaluation system for the value of ecological products was established, comprising 3 primary indices for provisioning, regulating and cultural services as well as 16 secondary indices, such as those for agricultural and forestry products.

For each indicator, a calculation of its biophysical valuation was first carried out (i.e., the volume of products or services provided by the ecosystem over a certain period of time was measured for each indicator). Following this, the monetary values of various ecosystem products and services were accounted for based on market pricing, alternative costs or simulated market methods. Once the value of each indicator was obtained, the values of the three primary indices were aggregated. In order to avoid large differences in magnitude due to the number of provisioning, regulating and cultural service sub-indices, which renders comparison difficult and may result in biases, this study normalised the value of these sub-indices to obtain the provisioning service index, regulating service index and cultural service index. Then, these figures were summed up to derive the composite GEP index using the following formula:

(1) $$I_{\mathrm{GEP}}=I_{\mathrm{EPV}}+I_{\mathrm{ERV}}+I_{\mathrm{ECV}}$$

where $I_{\mathrm{GEP}}$ is the composite GEP index, $I_{\mathrm{EPV}}$ is the provisioning service index, $I_{\mathrm{ERV}}$ is the regulating service index and $I_{\mathrm{ECV}}$ is the cultural service index.

#### 2.2.3. Analysis of Results

To better illustrate the spatial distribution characteristics of the evaluation results, LISA cluster analyses were performed on the results of the provisioning service index, regulating service index, cultural service index and composite GEP index separately. After the LISA cluster analyses, each county-level region was classified into “High-High”, “High-Low”, “Low-High” and “Low-Low” categories based on its own index value and the index value of the surrounding areas. The “High-High” category reflects that the index of the county-level region itself is high and the indices of the surrounding regions are also high; the “High-Low” category reflects that the index of the county-level region itself is high but the indices of the surrounding regions are low; the “Low-High” category that the index of the county unit itself is low but the indices of the surrounding regions are high; the “Low-Low” category reflects that the index of the county-level region itself is low and the indices of the surrounding regions are also low. The “Not Significant” category reflects that the spatial agglomeration characteristics of the county-level regions are not significant.

Furthermore, to explore the factors associated with the GEP evaluation results, a Pearson correlation analysis was performed to explore how the provisioning service index, regulating service index, cultural service index and composite GEP index correlate with economic levels and land use situations. The GDP was used to reflect the economic level of each county-level region, and the proportion of each land type was used to reflect its land use situation. Based on the results of the correlation analysis, the mechanisms behind the spatial distribution of various indices of the county-level regions were found and are discussed.

#### 2.2.4. Overall Technology Roadmap

**Figure 2:**
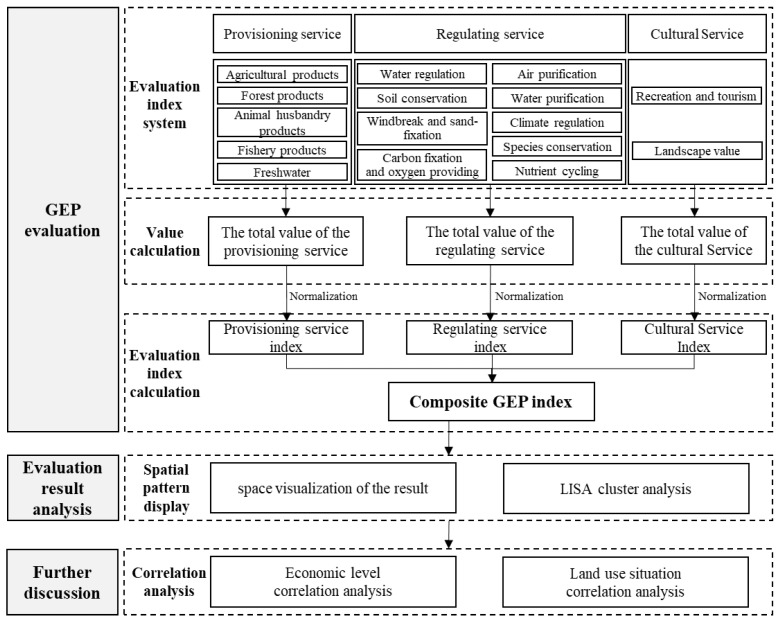
Technology Roadmap.

In this study, a comprehensive GEP evaluation system was constructed to evaluate the value of the ecological products of China’s county-level regions based on the existing GEP-related accounting system. The results were analysed after the evaluation, including the LISA clustering, to reflect the spatial distribution of the findings; a correlation analysis was also used to study the correlation between the results and the associated economic levels and land use situations. The overall roadmap is shown in Figure 2.

### 2.3. Data Sources

This study applies to massive geographic data covering the entire scope of the study. The basic geographical data, such as the administrative divisions used in the comprehensive evaluation of GEP in this study, were mainly sourced from the National Platform for Common Geospatial Information Services Geographic Information Public Service Platform (Tianditu), whereas the data used for calculating each indicator were extracted from public data. Among them, the indices of provisioning services mainly used statistical data of agricultural and fishery products from statistical yearbooks and bulletins, and land use and land cover change (LUCC) data originated from the Chinese Academy of Sciences’ Resource and Environmental Science Data Center. The indices of regulatory services drew on more diversified data, including LUCC data from the Resource and Environmental Science Data Centre, ecological monitoring data from meteorological, environmental, natural resources and other administrative departments and pricing data of oxygen production and sand treatment from water conservation and commodity price departments. Cultural service indices were extracted from tourism revenue statistics (from statistical yearbooks and bulletins), and land use data were extracted from the geospatial data cloud platform of the Computer Network Information Center at the Chinese Academy of Sciences.

In the later discussion section, the data of the economic level of county-level regions were obtained from statistical yearbooks and bulletins, and land resource data were taken from global landcover data (of 30 m precision) from the Ministry of Natural Resources of China and the National Geomatics Center of China.

## 3. Results

### 3.1. Provisioning Service Index

**Figure 3:**
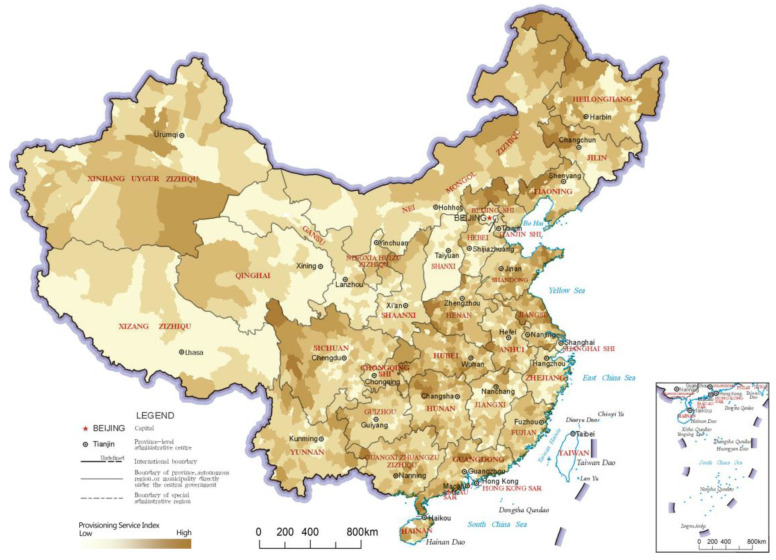
Distribution map of provisioning service indices of the county-level regions in China.

**Figure 4:**
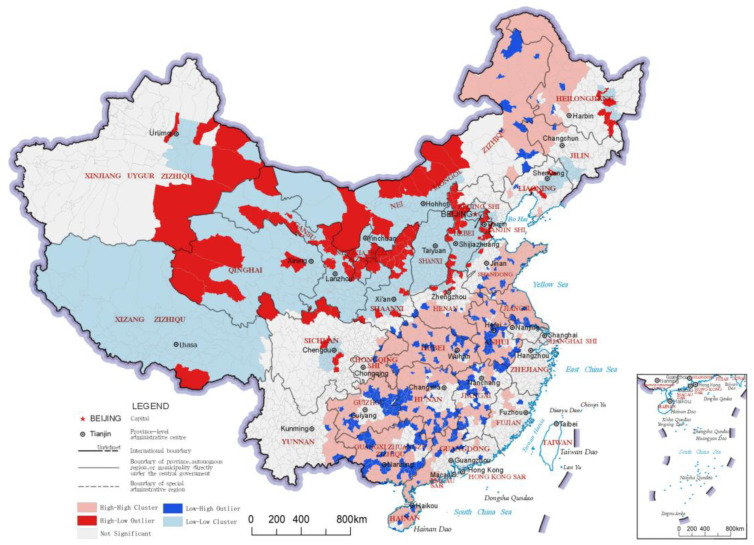
LISA cluster map of provisioning service indices of the county-level regions in China.

The provisioning service index reflects the effectiveness of the ecosystem in producing materials for market transactions, such as food, fishery products, timber and freshwater. The distribution results and LISA analysis results of the provisioning service indices of China’s county-level regions are shown in Figure 3 and Figure 4, respectively. In the LISA cluster analysis results, there were two main “High-High” clusters of the provisioning service index, one in northeastern China and the other in southeastern China. The northeastern cluster includes the northeastern part of Inner Mongolia and the northwestern regions of Heilongjiang and Jilin, which are cut across by the Greater Khingan Mountains and the Lesser Khingan Mountains. These mountainous areas are rich in forestry products. Southeast of the region of this northeastern cluster is the Northeast Plain, which is rich in food products produced with the vast amount of high-quality arable land, thus making the region very prominent for provisioning service value. At the same time, a large cluster of “High-High” category county-level regions was found in southeastern China, including eastern and southern Shandong, northern Jiangsu, southern Henan, northern Anhui, Hubei, northern Hunan, northern and eastern Chongqing, western and southern Guangxi, and northern Guangdong, which are dominated by plains and hilly areas that are more suitable for the production of various types of provisioning services. In addition, a large cluster of “Low-Low” category county-level regions was found in northwestern China, with several scattered “High-Low” category county-level regions located in the middle. These “High-Low” category county-level regions have good development of animal husbandry, and therefore, they performed better in the provisioning service index.

### 3.2. Regulating Service Index

**Figure 5:**
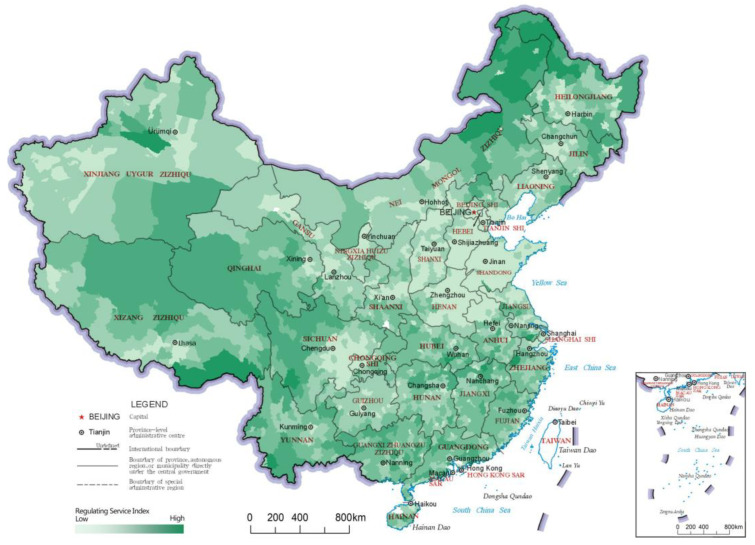
Distribution map of regulating service indices of the county-level regions in China.

**Figure 6:**
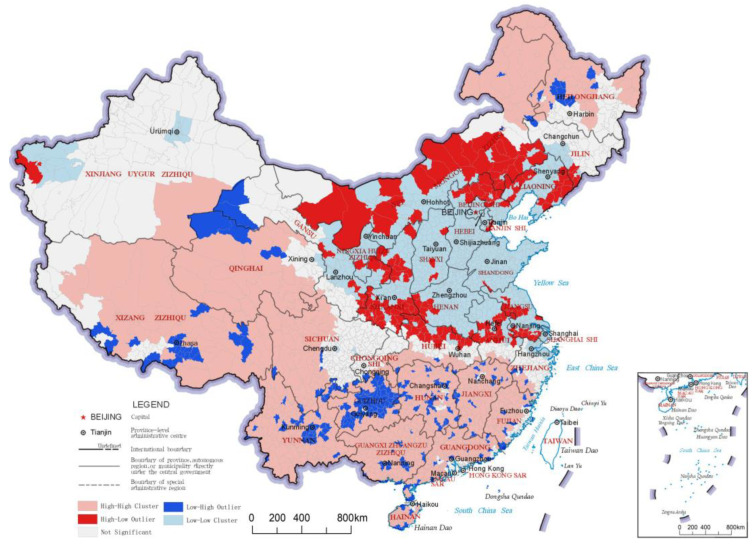
LISA cluster map of regulating service indices of the county-level regions in China.

The regulating service index reflects the ability of the ecosystem to improve people’s survival and quality of life, and it is an important reflection of ecological value. The distribution results and LISA analysis results of the regulating service indices of China’s county-level regions are shown in Figure 5 and Figure 6, respectively. In the results of the LISA clustering analysis, there were two main clusters of the “High-High” category county-level regions for the regulating service index. The first was northeastern China, including northeastern Inner Mongolia and northern Heilongjiang, which are crossed by the Greater Khingan Mountains and the Lesser Khingan Mountains ranges. This cluster’s forest resources play a role in carbon fixation and oxygen release, climate regulation and soil conservation, reflecting significant regulating services. The other cluster of “High-High” category county-level regions was found south of the Yangtze River and in a large area of the Qinghai-Tibetan Plateau. The area south of the Yangtze River has a humid climate with many hills, mountains and water bodies, which contributes to better ecosystem-regulating services. On the other hand, the Qinghai-Tibet Plateau area has many snow-capped mountains and glaciers, with services like water conservation, soil conservation, wind and sand control, carbon and oxygen fixation and biodiversity protection, thereby constituting an important ecological barrier in China. The northern part of the middle and lower reaches of the Yangtze River Plain and Inner Mongolia Plateau formed a number of clusters of “High-Low” category county-level regions due to their ecologically diverse and high-quality grassland resources. The “Low-Low” clusters in the regulating service index were mainly concentrated in the Loess Plateau and North China Plain; whereas the Loess Plateau has relatively lesser vegetation due to arid climate and soil erosion, the North China Plain has important production functions, as it has relatively concentrated arable land and low levels of ecological diversity.

### 3.3. Cultural Service Index

**Figure 7:**
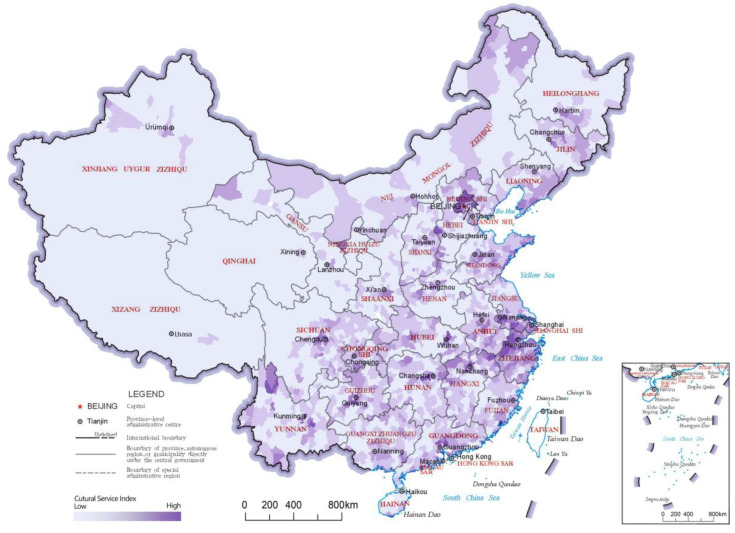
Distribution map of cultural service indices of the county-level regions in China.

**Figure 8:**
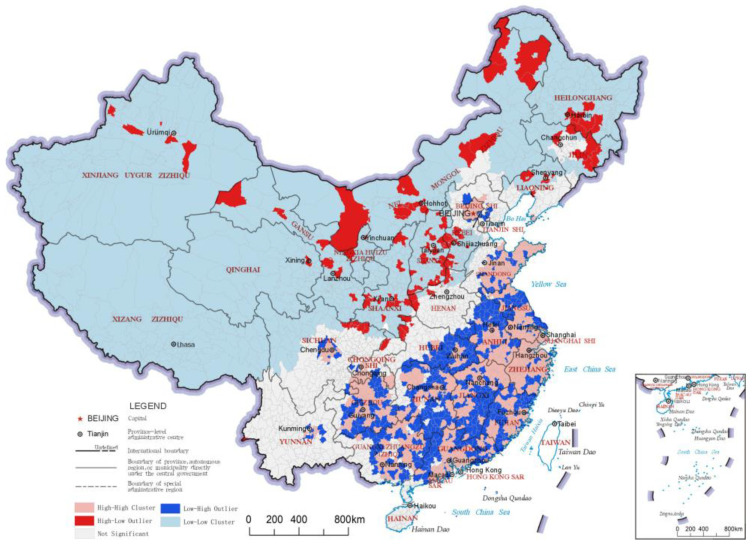
LISA cluster map of cultural service indices of the county-level regions in China.

The cultural service index reflects the non-material benefits people receive from the ecosystem, such as recreation, knowledge and aesthetic experiences. The distribution results and LISA analysis results of the cultural service indices of China’s county-level regions are shown in Figure 7 and Figure 8, respectively. As seen in the results of the LISA clustering, the clusters of “High-High” category county-level regions for the cultural service index comprised one large cluster and three small clusters. The large cluster was in southeastern China, including areas in eastern Shandong, southern Jiangsu, Zhejiang, southern Anhui, eastern Hubei, northern Hunan and southern Guangdong. Most county-level regions in this cluster have relatively good economic development levels and ecological resources. This catchment area was also interspersed with scattered “Low-High” category county-level regions, which are usually parts of the region with relatively low levels of economic development. In addition, three small “High-High” clusters formed around Beijing, Chengdu and Kunming, reflecting that the high value area of cultural service index has the characteristics of following the distribution of some large cities. In northern China, a large cluster of “Low-Low” county-level regions formed. Many county-level regions in this cluster underperform in terms of ecosystem cultural services, but there are some county-level regions that perform well in terms of ecosystem regulation services, such as those in the Greater Khingan Mountains and those in the Lesser Khingan Mountains ranges and the Qinghai-Tibet Plateau, where high-quality ecological resources are not transformed into cultural services such as recreation.

### 3.4. Composite GEP Index

**Figure 9:**
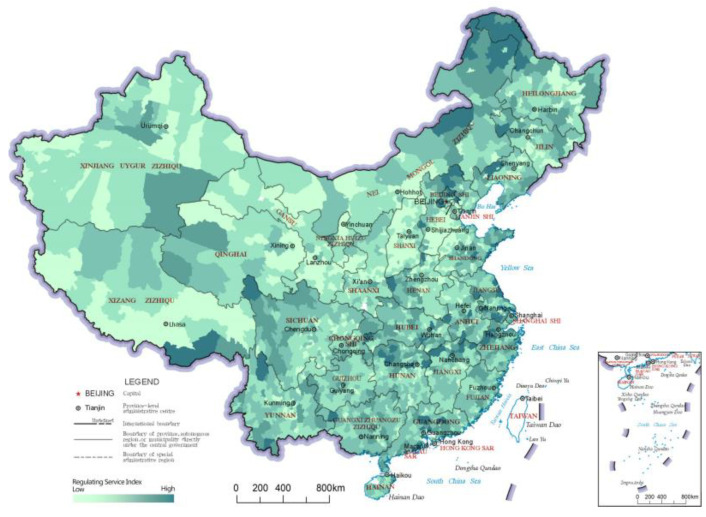
Distribution map of composite GEP indices of the county-level regions in China.

**Figure 10:**
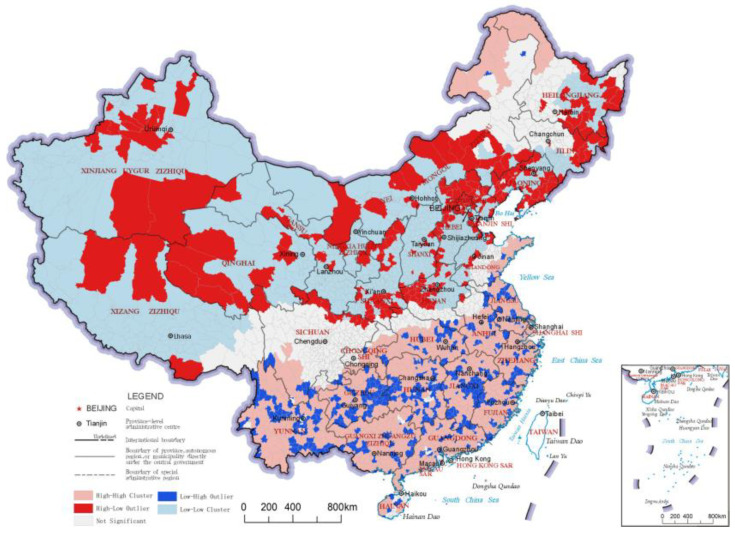
LISA cluster map of composite GEP indices of the county-level regions in China.

The composite GEP index is a combination of three sub-indices: provisioning service, regulation service and cultural service, reflecting the overall effectiveness of the county-level regions’ ecological product transformation. The distribution results and LISA analysis results of composite GEP index of China’s county-level regions are shown in Figure 9 and Figure 10, respectively. With LISA clustering, two clusters were found in the “High-High” category of the GEP index. One large cluster is located in southern China, including a large area south of the Yangtze River and Jiangsu, eastern Shandong, and southwestern Sichuan. More county-level regions in this cluster perform better in terms of provisioning service, regulatory service and cultural service, thereby exhibiting higher effectiveness in the value transformation of ecological products. Another “High-High” cluster was found in northeastern Inner Mongolia and northwestern Heilongjiang in northeastern China (the northern part of the Greater Khingan Mountains), areas which perform well in several sub-indices. Many clusters of “High-Low” county-level regions were in the border area of Xinjiang, Tibet and Qinghai in western China, where there is a diversity of landforms. The region is crossed by the Tianshan Mountains, Kunlun Mountains and Qilian Mountains, including the Qinghai-Tibetan Plateau, Qaidam Basin and Tarim Basin, and it also includes the headwater areas of the Yangtze River, Yellow River and Lancang River, making it a concentration of ecological value transformation in northwest China. The “Low-Low” GEP index clusters were mainly in northern and northwestern China, which mainly include two types of areas: first, areas with little topographic variation that are relatively water-scarce, such as the North China Plain and Loess Plateau, where county-level regions usually lacked strength in all three sub-indices; second, areas with high topographic variation and little water scarcity but with unique climatic conditions and less transport accessibility, such as the Gangdis Mountains in southern Tibet and the Himalayan regions, which performed well in the regulating services sub-index but not in material goods and cultural services (resulting in poor performance in the composite GEP index).

## 4. Discussion

So far, many scholars have carried out research with ecological product value evaluation, though most of them researched at a city or region scale [15,16,17,18,19]. These studies have provided useful explorations for GEP technical methods, but they have not conducted a comprehensive study of the overall spatial patterns and underlying mechanisms on a larger scale. In 2017, Ma used the GEP method to evaluate China’s terrestrial ecosystem value and analysed the spatial pattern at the province level [21]. This study pointed out that the spatial differences of China’s GEP are relatively large, among which the total values of GEP in Inner Mongolia and Heilongjiang were the highest, followed by Tibet, Sichuan, Guangdong and Yunnan. Compared with Ma’s research, we evaluated China’s ecological product value at a county-level, improving the fineness and granularity of the spatial pattern. We also optimized the evaluation indices system, adding some indices such as species conservation, nutrient cycling, landscape value, etc. The new system enables the ecological value of some ecological high-quality urban areas and woodlands to be sufficiently shown. By normalising the value of these sub-indicators to obtain GEP indices, this study avoids the sub-item index having too much influence on the evaluation results due to the large number of indices, and the results reflect the relative relationship between GEP’s spatial distribution and factors more accurately.

Ma pointed out that different land uses have different impacts on ecological products’ value. For example, the value of ecological products in territorial waters and forests was the highest. In addition, some scholars have found that the level of economic development also has an impact on ecological products’ value [4,34,35]. The economically developed regions have higher demand for ecological products and services. This means that, in addition to the characteristics of ecological resources, ecological product value is impacted by a complex multi-factor mechanism.

Therefore, further discussion about the correlations between GEP indices and other factors was carried out. Pearson correlation analysis was used to study the correlations between the GEP indices of China’s county-level regions and their economic levels (reflected using county-level region’s GDP) and land use situations (reflected by the proportion of various types of land areas in the county-level region) (Table 2).

The provisioning service index of the county shows the strongest positive correlation with the proportion of arable land area producing food. The main function of arable land area is to produce agricultural ecological products, and the value of material products belongs to its direct use value [36]. It is followed by the area of water producing fishery products and supplying fresh water as well as the area of woodland producing forestry products. Regarding the nationwide spatial distribution of the provisioning service index, areas with high provisioning service indices were concentrated in the northeastern and southeastern regions of China, with the northeastern region being relatively rich in arable land and woodland resources, and the southeastern region being relatively rich in arable land, woodland or water bodies. At the same time, the provisioning service indices of county-level regions had a strong negative correlation with the proportion of artificial surfaces in county-level regions. In fact, the high level of urban development in county-level regions means a smaller proportion of ecological and agricultural space for producing provisioning services, which usually leads to a relatively low level of transformation into provisioning services. The topographic and climatic conditions around coastal or large cities in southeastern China are not inferior to those in non-coastal areas in southeastern China, but coastal areas have not become areas with high provisioning service indices. This is mainly because these areas are usually more urbanised and have a large proportion of artificial surface area, which always means having fewer strengths to provide provisioning services.

The regulating service index of the county-level region has a significant positive correlation with the proportion of woodland areas in the county-level region. Woodland provides a variety of ecological services, including water regulation, water purification and species conservation [34]. It is followed by the proportions of grassland area, water area, glacier area, shrubland area and wetland area. The value of ecosystem-regulating services is positively correlated with the proportion of ecological land, such as woodlands, grasslands, water bodies and glaciers. The spatial distribution of regulating service indices in China indicates that northeastern and southern China and the Qinghai-Tibetan Plateau are the areas where high values of regulating services are concentrated. Among them, northeastern and southern China are rich in woodlands or water bodies, whereas the Qinghai-Tibetan Plateau in western China has more grasslands and glaciers. The regulating service index was negatively correlated with the proportion of man-made areas, the proportion of arable land, and GDP, reflecting the fact that if the urbanisation level of a county-level region is relatively high, or if the role of food production is relatively significant, the transformation into ecological regulating services is usually relatively low. Areas with low values of regulation service indices were mainly concentrated in the North China Plain, where there are relatively low proportions of woodland and water areas and relatively high proportions of arable land. Therefore, land use structure can result in limited regulation services.

The county’s cultural service index had the strongest positive correlation with GDP. Existing studies also show that economic growth provides strong support for tourism development [37]. It is followed by the proportions of water area, man-made and woodland areas. Hence, ecosystem cultural services have a strong positive correlation with the level of local economic development and the condition of natural resources such as local water bodies and woodlands. With regards to the spatial distribution of China’s cultural service index, areas with high cultural service indices are in southeastern China and around some large cities, which reflects the correlation between cultural services and economic development. At the same time, the cultural service index has a negative correlation with the proportion of grassland area and bare land. County-level regions with more grassland resources are usually relatively deficient in cultural services, whereas county-level regions with more bare land indicate that the local land is rough or that it is more difficult to cultivate high-quality vegetation due to climate; therefore, this makes it difficult to transform ecosystem products into services such as culture and leisure. A large part of the northeast is rich in grassland resources, but ecological resources have not been transformed into cultural services, whereas the northwest, which has a large distribution of bare land, faces the same issue.

The composite GEP indices of the county-level regions and the proportions of woodland and water areas showed a significant positive correlation as well as a moderate correlation with GDP level. The proportions of water area and forest resources in land use were positively correlated with transformations into provisioning service, regulating services and cultural services, and therefore, they had a strong positive correlation with the composite GEP index. GDP had the strongest positive correlation with the cultural service index, resulting in positive correlation with the composite GEP index. For the spatial distribution of China’s GEP index, the large area in the south of China and the area in the northeast of China formed two high-value clusters. The southern region is rich in water area and woodland resources and has a high level of economic development, whereas the northeast region has a very prominent advantage in terms of woodland resources, which promotes the transformation of ecological product value.

Ma (2017) proposed that water and woodland contribute the most to ecological product value [21]. In our exploration, it can be seen that both the proportion of woodland area and the proportion of water area have strong positive correlations with various indices of GEP. Woodland areas are positively correlated with various indices of GEP evaluation, among which they have the strongest positive correlation with the regulating service index. Some scholars found that woodlands play a very important role in windbreak and sand fixation, carbon fixation, biodiversity maintenance, etc. [37] At the same time, woodlands provide material products such as trees and crops planted under the trees [38], and woodlands with convenient transportation can also provide landscape viewing [39]. Second, water areas are also positively correlated with various indices of the GEP evaluation, with the strongest positive correlation being with the cultural service index. Water usually has an important landscape viewing function, but it also can provide fresh water and fishery products and play an important role in climate regulation, carbon fixation and other functions. Scholars have also found that increasing of the area of woodland or water is likely to effectively enhance ecological product value [40,41]. At the same time, this analysis also found that some elements are positively correlated with some indices of GEP evaluation, whereas they are negatively correlated with other indices. For example, GDP has a strong positive correlation with the cultural service index, whereas it has a negative correlation with the cultural service index and regulating service index. Research by other scholars also showed that places with better economic development usually gather more people, and people with higher living standards will have higher demand for ecosystem cultural services to promote the ecological space to provide cultural service [42]. However, many economically developed areas with high degrees of urbanization have long focused on economic development instead of ecological development, and thus, they turn out to be relatively inferior in providing provisioning services and regulating services [43,44]. Therefore, these places should balance economic development and ecological space improvement [4]. In addition, from this analysis, it was found that some elements only have general or weak correlations with some indices, and that the correlation with other indices is not significant. For example, shrubland only has a weak correlation with the regulating service index, and its correlation with other indices is not significant. Shrublands usually have sparse vegetation and low tree heights with less prominent regulation functions, low efficiency of material products and low landscape appreciation. Therefore, to improve the ecological product value quickly and efficiently, increasing shrubland may not be the priority choice. If the climate and geographical conditions allow, it will be more efficient to moderately increase the area of woodland and water.

## 5. Conclusions

By referring to many standard criteria and research practices in GEP evaluation indices and methods, this study built a GEP evaluation system for China’s county-level regions, obtained three major sub-indices—provisioning service index, regulating service index and cultural service index—as well as the composite GEP index, and carried out the spatial pattern analysis of the GEP index and correlation analysis of the GEP index with other factors.

The analysis shows that the GEP indices in China’s county-level regions have significant spatial distribution differences, and the factors associated with each index are different because the values of ecological products are meaningful in different ways. In terms of the provisioning service index, northeastern and southeastern China are outstanding regions, relying on their advantages in resources such as arable land, water area and woodlands to promote the transformation of regional resources into agricultural products, fishery products and forestry products. In terms of the regulating service index, northeastern China, a large area south of the Yangtze River and the Qinghai-Tibet Plateau are the areas with more concentrated regulating services. The northeastern part of China, with its vast and high-quality woodland resources, the large area south of the Yangtze River, with its extensive woodlands and water area, and the Tibetan Plateau, with its abundant grasslands and glaciers, enable important functions such as carbon fixation and oxygen release, soil conservation and biodiversity maintenance. In terms of the composite GEP index, the regions with high value usually also performed well in several sub-indices, including the northeastern and southern regions and the northwestern region. These areas are usually rich in ecological resources, such as water areas and woodlands, which promote transformation into material goods and regulatory services by virtue of their resource advantages, whereas large areas in southern China have also promoted the transformation into cultural services through having more developed economies, which is ultimately a manifestation of the advantages of ecological product value.

The effectiveness of ecological value transformation is the result of various complex factors, and the analysis of numerous samples at the national level reveals that it is related to both the natural resource base and the level of economic development. For specific geographical spaces, in addition to the resource base and the level of economic development, the spatial structure, resource allocation, industrial orientation and construction works of county-level regions also affect the level of value of local ecological products. Therefore, plans, policies and projects that affect the spatial use and direction of development in county-level regions usually become important tools to promote the transformation of ecosystem values. At the same time, the results of the GEP evaluation also provide a reference point for the formulation of these plans, policies and projects. For example, GEP evaluation contributes to urban spatial planning, providing reference for functional land zones, spatial layout and element control. Specifically, the GEP evaluation results clearly reveal the advantages, disadvantages and spatial distribution of the ecological product value. Thus, according to the GEP evaluation results and the land use and economic conditions, spatial planning can form a more effective spatial strategy to better achieve the planning objectives. On one hand, spatial planning can promote the adjustment of the structure of land use. For example, for places with low regulating service indices and low proportions of woodland areas, which are the most relevant to the regulating service index, the adjustment of land use structure can be considered in spatial planning, such as in determining the appropriate portion of idle land to restore to woodland, the ecological restoration of existing polluted woodlands and water, etc. On the other hand, spatial planning can promote the release of the value of ecological products in existing land, especially for places where the GEP evaluation index results are not prominent but the factors with high correlation have advantages; this reflects the lack of effective transformation of ecological product value in these places, in which case it is necessary to propose specific paths for the transformation of ecological product value. For example, for places with high proportions of arable land area but low provisioning service indices, spatial planning can promote the improvement of farming technology and machinery to improve the production efficiency of agricultural products; thus, it would strengthen the brand effect of agricultural products to improve the value of agricultural products. What’s more, for places with high-quality woodland and water and the advantage of high GDP or being adjacent to economically developed large cities, spatial planning can optimise the traffic connections between ecological spaces and urban centres and improve the tourism service infrastructure in ecological spaces in order to improve the cultural service value of ecological spaces. It can be concluded that the GEP evaluation results can be obtained by using geospatial technologies, which can help form targeted strategies to enhance the value of ecological products in spatial planning.

The deficiency of this study is that the evaluation system does not cover all valuable ecological resources due to the difficulty of data acquisition and immature technical methods. For example, the value of ice, snow and coastal zones have not been evaluated. The follow-up research will supplement some relevant indices to measure the value of marine resources, ice, snow and coastal zones and other potential ecological resources by optimizing data and technology. The follow-up study may also carry out a more detailed evaluation of the ecological product value of some typical regions and put forward suggestions to improve the value of ecological products.

## Figures and Tables

**Table 1:** List of ecosystem services value evaluation indices in other relevant studies.

Ecosystem Services	United Nations Millennium Ecosystem Assessment (MA)	European Union The Economics of Ecosystems and Biodiversity (TEEB)	United Nations Experimental Ecosystem Accounting (EEA)	Technical Guide for Gross Terrestrial Ecosystem Product (GEP) Calculation
Provisioning services	Food Fresh water Fuelwood Fibre Biochemicals Genetic resources	Food Water Raw materials Genetic resources Medicinal resources Ornamental resources	Water Materials Energy Other provisioning services	Agricultural products Forest products Animal husbandry products Fishery products Ecological energy Others
Regulating services	Climate regulation Disease regulation Water regulation Water purification	Air purification Climate regulation Disturbance prevention or moderation Regulation of water flows Waste treatment Erosion prevention Maintaining soil fertility Pollination Biological control	Remediation and regulation of biophysical environment Flow regulation Regulation of physicochemical environment Regulation of biotic environment	Water conservation Soil conservation Windbreak and sand-fixation Coastal zone protection Flood water storage Carbon fixation Oxygen providing Air purification Water purification Pollination Climate regulation Species conservation
Cultural services	Spiritual and religious Recreation and ecotourism Aesthetic Inspirational Educational Sense of place Cultural heritage	Aesthetic information Recreation and tourism Inspiration for culture, art and design Spiritual experience Information for cognitive development	Physical or experiential use of ecosystems Intellectual representations of ecosystems	Recreation and tourism Landscape value
Supporting services	Soil formation Nutrient cycling Primary production	Lifecycle maintenance Gene pool protection	/	/

**Table 2:** Correlation between GEP indices and economic level and land use situation factors.

Classification	Elements	Provisioning Service Index	Regulating Service Index	Cultural Service Index	Composite GEP Index
Economic level	GDP	−0.015	−0.133 **^,1^	0.394 **	0.196 **
Land use situation	Proportion of water area	0.109 **	0.092 **	0.217 **	0.223 **
	Proportion of woodland area	0.104 **	0.404 **	0.121 **	0.262 **
	Proportion of grassland area	−0.025	0.181 **	−0.199 **	−0.078 **
	Proportion of artificial surface area	−0.302 **	−0.397 **	0.143 **	−0.196 **
	Proportion of wetland area	0.086 **	0.067 **	−0.036	0.044 *
	Proportion of bare land area	−0.043 *	0.006	−0.139 **	−0.107 **
	Proportion of shrubland	−0.007	0.069 **	0.010	0.025
	Proportion of arable land area	0.115 **	−0.345 **	−0.052 **	−0.086 **
	Proportion of glacier area	−0.049 **	0.086 **	−0.083 **	−0.049 **

^1^ In the table, ** represents a significant correlation at the 0.01 level (two-sided), and * represents a significant correlation at the 0.05 level (two-sided).

## Data Availability

Not applicable.
